# Ultrasound Diagnosis of Duodenal Web

**DOI:** 10.5334/jbsr.2819

**Published:** 2022-05-23

**Authors:** Nando De Vulder, Marie-Sofie Walgraeve

**Affiliations:** 1 AZ Sint-Jan Brugge, BE

**Keywords:** Duodenal web, neonate, ultrasound

## Abstract

**Teaching Point:** Adequate dynamic evaluation of the duodenum by ultrasound can aid in the differential diagnosis of high intestinal obstruction.

## Case History

A seven-day-old boy was admitted to the neonatal intensive care unit due to feeding intolerance with persistent vomiting and poor weight gain. His medical history consisted of postnatal amoxicillin-amikacin administration for prolonged ruptured membranes and postnatal tight glycaemic conditions for which supplemental feeding was started in addition to breastfeeding. He started vomiting two days after birth, with increasing intensity until he vomited after and between each feeding. On admission clinical examination showed a malnourished baby with icteric skin and poor peripheral circulation. Abdominal ultrasound was requested as a general screening examination.

**Figure 1:**
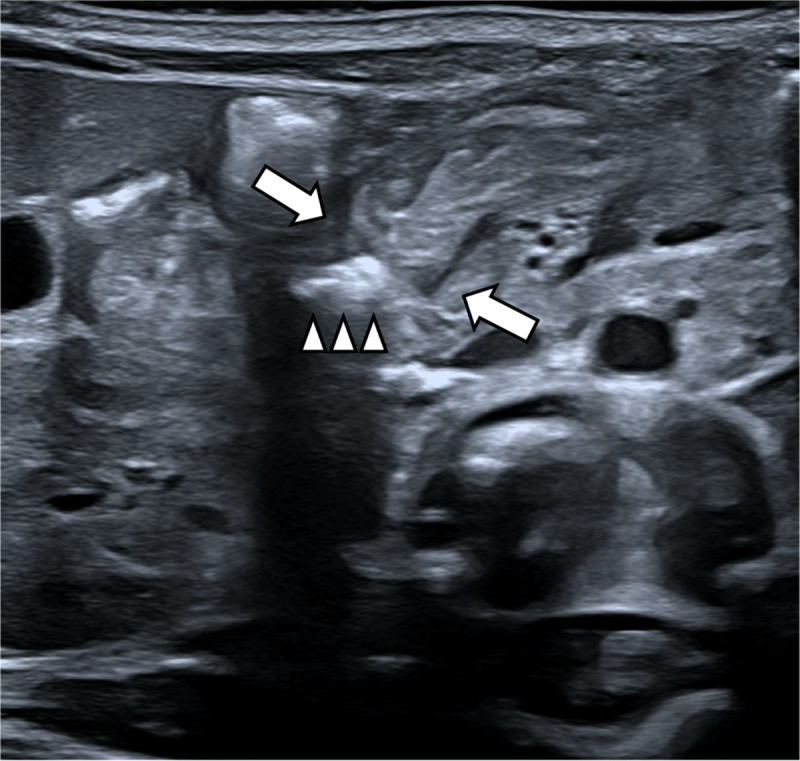


**Figure 2:**
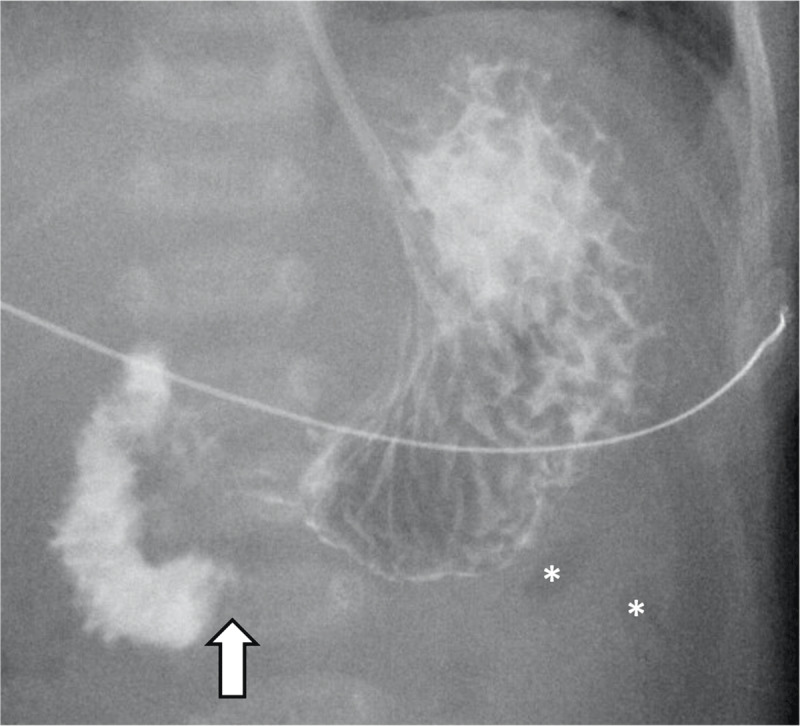


**Figure 3:**
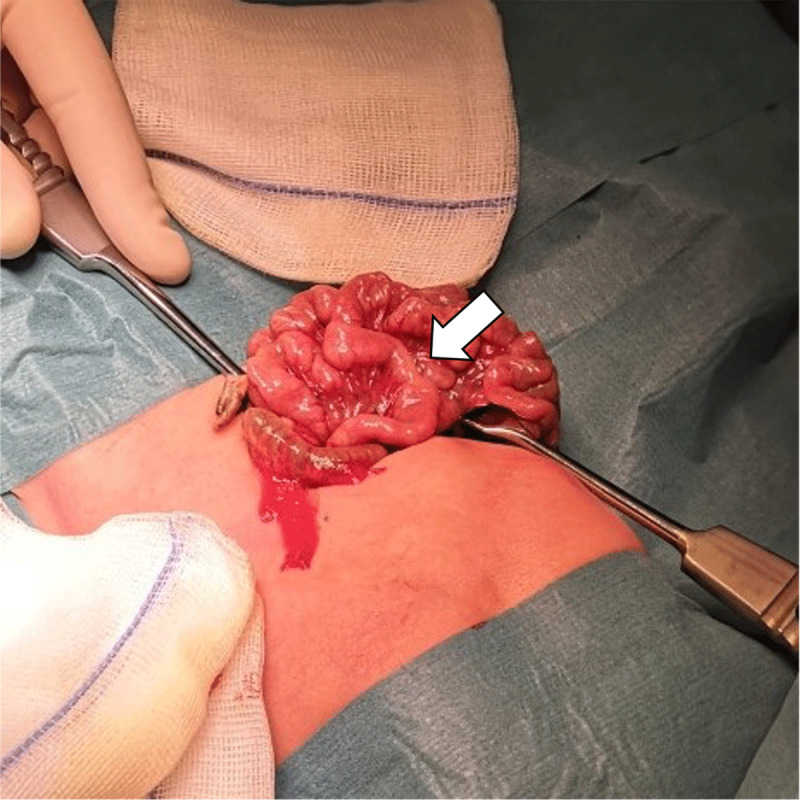


Ultrasound showed a normal relationship of the mesenteric vessels, normal location of the duodenojejunal flexure and no pancreatic abnormalities. However, a focal duodenal stenosis with pseudo-invagination image was noted at the level of the duodenal D3 segment (arrows in Figure 1). Stasis of intraluminal air could be seen up to this calibre narrowing (arrowheads in Figure 1). The duodenum distal to this stenosis seemed to be normal. Possible diagnosis of a duodenal web was suggested, and additional Barium swallow procedure with Ultravist 300 was requested. On this examination, the passage problem was confirmed (arrow in Figure 2) with clear tapering of the duodenal lumen at the level of D3. Also, there was only a limited amount of air in the distal small intestine (asterisks in Figure 2). Clinical and imaging findings were all in keeping with diagnosis of a duodenal web. After three days of supportive treatment, a laparotomy was performed, confirming duodenal web with a focal narrowing at D3-level of the duodenum (arrow in Figure 3). Subsequently, a duodeno-duodenostomy was performed, bypassing the stenosis. After surgery, enteral feeding was gradually induced. The patient was discharged 14 days post-surgery in good health.

## Comment

The differential diagnosis of high gastrointestinal obstruction in neonates consists of gastric outlet obstruction, duodenal obstruction, jejunal obstruction or malrotation with secondary midgut volvulus. Due to embryologically varying degrees of lack of recanalisation, congenital duodenal obstruction represents a broad spectrum, ranging from complete atresia to stenosis or duodenal web. The diagnosis can usually be made on conventional radiography by the presence of a double-bubble sign and absence of air distal to the duodenum. However, in cases of incomplete duodenal obstruction, variable amounts of air distal to the stenosis may be found and the initial image on conventional radiography may look less typical [1].

This case illustrates that ultrasound can not only aid in the diagnosis of high intestinal obstruction by ruling out malrotation and midgut volvulus, but also by suggesting duodenal obstruction. Particularly in non-typical cases adequate dynamic evaluation of the duodenum could expedite diagnosis and guide appropriate management.
